# Genotype-phenotype correlation in neuronal migration disorders and cortical dysplasias

**DOI:** 10.3389/fnins.2015.00181

**Published:** 2015-05-21

**Authors:** Mitsuhiro Kato

**Affiliations:** Department of Pediatrics, Yamagata University Faculty of MedicineYamagata, Japan

**Keywords:** lissencephaly, heterotopia, polymicrogyria, tubulinopathy, interneuronopathy, *LIS1*, *DCX*, *ARX*

## Abstract

Neuronal migration disorders are human (or animal) diseases that result from a disruption in the normal movement of neurons from their original birth site to their final destination during early development. As a consequence, the neurons remain somewhere along their migratory route, their location depending on the pathological mechanism and its severity. The neurons form characteristic abnormalities, which are morphologically classified into several types, such as lissencephaly, heterotopia, and cobblestone dysplasia. Polymicrogyria is classified as a group of malformations that appear secondary to post-migration development; however, recent findings of the underlying molecular mechanisms reveal overlapping processes in the neuronal migration and post-migration development stages. Mutations of many genes are involved in neuronal migration disorders, such as *LIS1* and *DCX* in classical lissencephaly spectrum, *TUBA1A* in microlissencephaly with agenesis of the corpus callosum, and *RELN* and *VLDLR* in lissencephaly with cerebellar hypoplasia. *ARX* is of particular interest from basic and clinical perspectives because it is critically involved in tangential migration of GABAergic interneurons in the forebrain and its mutations cause a variety of phenotypes ranging from hydranencephaly or lissencephaly to early-onset epileptic encephalopathies, including Ohtahara syndrome and infantile spasms or intellectual disability with no brain malformations. The recent advances in gene and genome analysis technologies will enable the genetic basis of neuronal migration disorders to be unraveled, which, in turn, will facilitate genotype-phenotype correlations to be determined.

## Introduction

The characteristic six-layered neocortex in the human brain is formed by two types of neuron, projection neurons and interneurons, which migrate from their birth places, such as the ventricular zone and ganglionic eminence, respectively. Neuronal migration disorders are human (or animal) diseases that result from the disruption of normal movement of neurons from their original birth site to their final destination during early development. As a consequence, the neurons remain somewhere along their migratory route, their location depending on the pathological mechanism and its severity. Many genes have been found to be responsible for neuronal migration disorders, such as *LIS1* and *DCX* in classical lissencephaly spectrum, *TUBA1A* in lissencephaly with cerebellar hypoplasia, *ARX* in X-linked lissencephaly with abnormal genitalia (XLAG), *FLNA* and *ARGEF2* in periventricular heterotopia, *FCMD* and glycosylation-related genes, such as *POMT1, POMT2, POMGNT1, POMGNT2, FKRP, LARGE, TMEM5, POMK, ISPD, GMPPB, B3GNT1*, and *B3GALNT2* in cobblestone dysplasias, *GPR56, SRPX2*, and some tubulin-related genes, e.g., *TUBA8, TUBB2B*, and *TUBB3*, in polymicrogyria (Kato and Dobyns, 2003; Vuillaumier-Barrot et al., 2012; Buysse et al., 2013; Stevens et al., 2013; Fry et al., 2014). Recently, we found that mutations in *COL4A1*, which encodes type IV collagen alpha 1 subunit, cause schizencephaly accompanied by polymicrogyria in the adjacent cortex of the transmantle cleft as well as focal cortical dysplasia (Yoneda et al., 2013). Historically, brain malformations including neuronal migration disorders have been classified based on a postmortem examination. The advancement and spread of neuroimaging techniques, particularly magnetic resonance imaging (MRI), make it easier to find out many types of brain malformations, but make it more complicated to classify them. Moreover, the unveiling of responsible genes for brain malformations has changed the classification scheme and causes most neuroscientists and even physicians trouble to follow it. Here, I review the clinical manifestation of neuronal migration disorders, focusing mainly on lissencephaly, in terms of genotype-phenotype correlations.

## Lissencephaly spectrum: classical lissencephaly to subcortical band heterotopia

Lissencephaly is classified as a spectrum of disorders caused by widespread abnormal transmantle migration, ranging from classical lissencephaly (agyria or pachygyria) to subcortical band heterotopia or double-cortex syndrome (Barkovich et al., 2012). Classical lissencephaly is characterized by a smooth (*lissos* in Greek) brain surface with a decreased number of sulci and wide gyri. Mutations in *LIS1*, located on chromosome 17p13.3, or *DCX* on Xq23 are the main cause for classical lissencephaly (Table 1) (Kato and Dobyns, 2003). Mutations in *DCX* are causative for classical lissencephaly in male individuals and subcortical band heterotopia in female individuals. A combination of a severity grading scale [the most severe form, Grade 1 (total agyria) to the mildest form, Grade 6 (subcortical band heterotopia) via the intermediate forms comprised of a combination of agyria, pachygyria, and subcortical band heterotopia] and an anterior or posterior gradient scale is useful to predict the causative gene for lissencephaly spectrum (Kato and Dobyns, 2003). For instance, mutations of *LIS1, ARX*, or *TUBA1A* result in a posterior more severe than anterior gradient, while mutations of *DCX* or *RELN* lead to an anterior more severe than posterior gradient. LIS1 participates in cytoplasmic dynein-mediated nucleokinesis, somal translocation, and cell motility (Smith et al., 2000) as well as mitosis or neurogenesis and chromosomal segregation (Faulkner et al., 2000). DCX is a microtubule-associated protein and is involved in microtubule polymerization and stabilization (Gleeson et al., 1999). Missense mutations in *DCX* responsible for lissencephaly spectrum are mainly located in two tandem repeats (N-terminal or C-terminal doublecortin domains), which bind to microtubules or free tubulin and other components (Friocourt et al., 2005), respectively.

**Table 1:** **Clinical features of gene mutations causing cortical disruptions**.

**Gene**	**Locus**	**Inheritance mode**	**LIS**	**HET**	**PMG**	**MIC at birth**	**ACC**	**PCH**	**Brain**	**Other findings**
*LIS1~YWHAE*	17p13.3	AD	+						Total agyria (Figure-of-8 appearance)	Characteristic face and MCA
*LIS1*	17p13.3	AD	+	+, rare					Agyria to subcortical band HET, mainly pachygyria in anterior and agyria in posterior	
*DCX* (male)	Xq23	XL	+	+					LIS. Subcortical band HET due to somatic mosaic mutation.	
*DCX* (female)	Xq23	XL	+, rare	+					Subcortical band HET	
*TUBA1A*	12q13.12	AD	+	+, rare	+, rare	+	+	+	MIC, agyria to subcortical band HET, PMG, PCH, ACC	
*TUBA8*	22q11	AR			+		+		PMG, agenesis or hypogenesis of the corpus callosum, dysmorphic brainstem	Optic nerve hypoplasia
*TUBB2A*	6p25.2	AD						+	Mild PCH	
*TUBB2B*	6p25.2	AD			+	+		+	MIC, PMG, dysmorphic basal ganglia, PCH, dysmorphic brainstema	CFEOM
*TUBB3*	16q24.3	AD			+			+	PMG, gyral disorganization, dysmorphic basal ganglia, PCH	CFEOM
*TUBB*	6p21.33	AD		+	+	+	+		MIC, focal band HET or PMG, dysmorphic basal ganglia, abnormal corpus callosum	Microophthalmia
*TUBG1*	17q21.2	AD	+	+					Posterior dominant lissencephaly, dysmorphic corpus callosum	
*ARX* (male)	Xp22.13	XL	+				+		Posterior dominant LIS with ACC and dysmorphic basal ganglia	Hypoplastic genitalia, diarrhea
*ARX* (female)	Xp22.13	XL					+		ACC in half of the cases	
*RELN*	7q22.1	AR	+					+	Anterior dominant diffuse pachygyria with PCH	
*VLDLR*	9p24.2	AR	+					+	Diffuse pachygyria with PCH	
*MCPH1*	8p23.1					+			MIC	
*WDR62*	19q13.12	AR	+	+	+	+	+		MIC, pachygyria, PMG, or subcortical band HET, abnormal corpus callosum	
*NDE1*	16p13.11	AR				+	+		MIC, simplified gyral pattern, ACC	
*COL4A1*	13q34	AD, low penetrance							Porencephaly, schizencephaly, focal cortical dysplasia	Myopathy, hematuria, anemia

*ACC, Agenesis of the corpus callosum; CFEOM, Congenital fibrosis of the extraocular muscle; HET, heterotopia; LIS, classical lissencephaly or agyria/pachygyria; MCA, multiple congenital anomalies; MIC, microcephaly; PCH, pontocerebellar hypolasia; PMG, polymicrogyria*.

**Figure 1:**
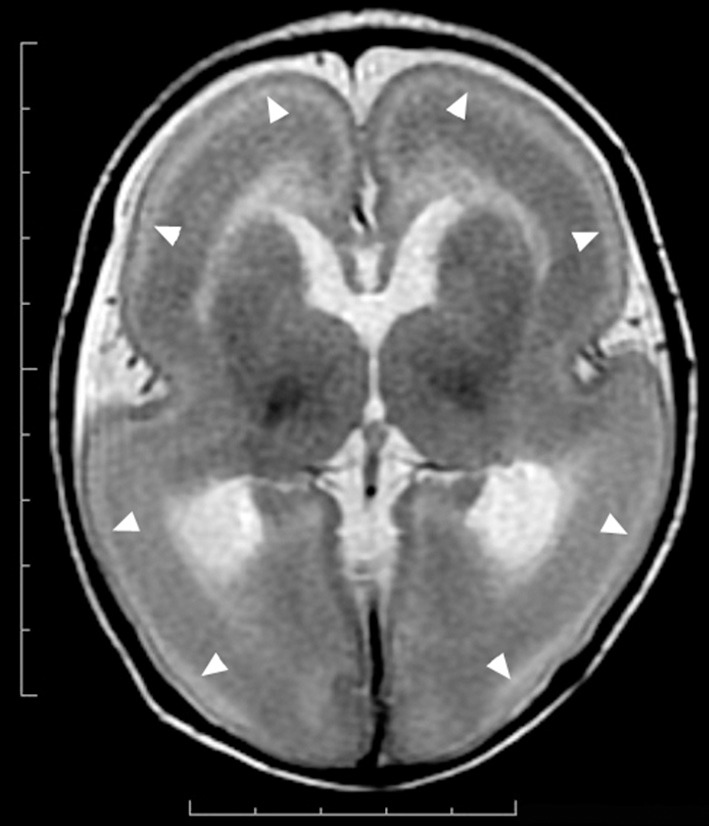
**Complete agyria in a** ***DCX*** **mutation patient (Grade 1 on the severity scale)**. T2-weighted axial MRI image. Wide shallow sylvian fissures create a figure-of-eight appearance. The thickness of the cortex is over 10 mm. A high-intensity (white) line (arrow heads) beneath the cerebral surface is consistent with a cell sparse layer of the four-layered cortex.

**Figure 2:**
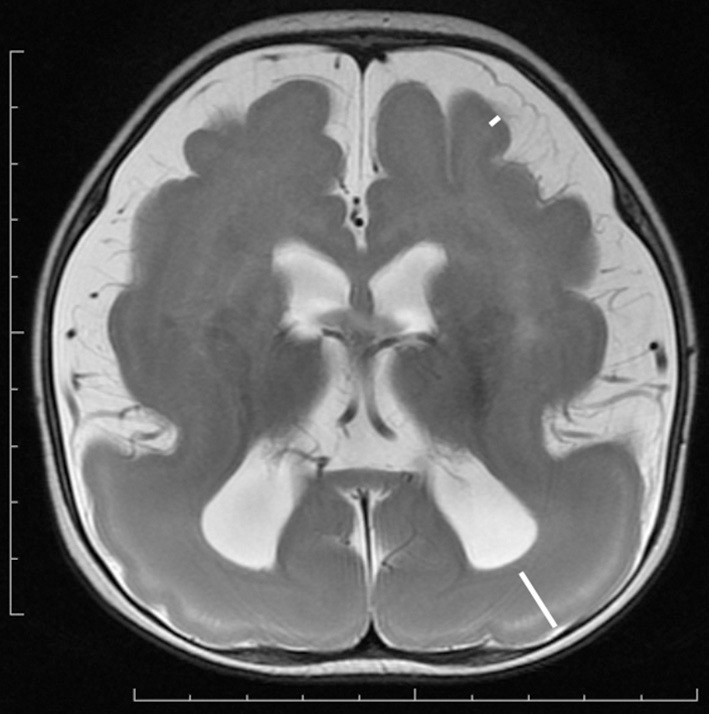
**Anterior pachygyria and posterior agyria in a** ***LIS1*** **mutation patient (Grade 3 on the severity scale)**. T2-weighted axial MRI image. Note the difference in the width of gyri, the depth of sulci and the thickness of the cortex (bars) between anterior and posterior regions.

**Figure 3:**
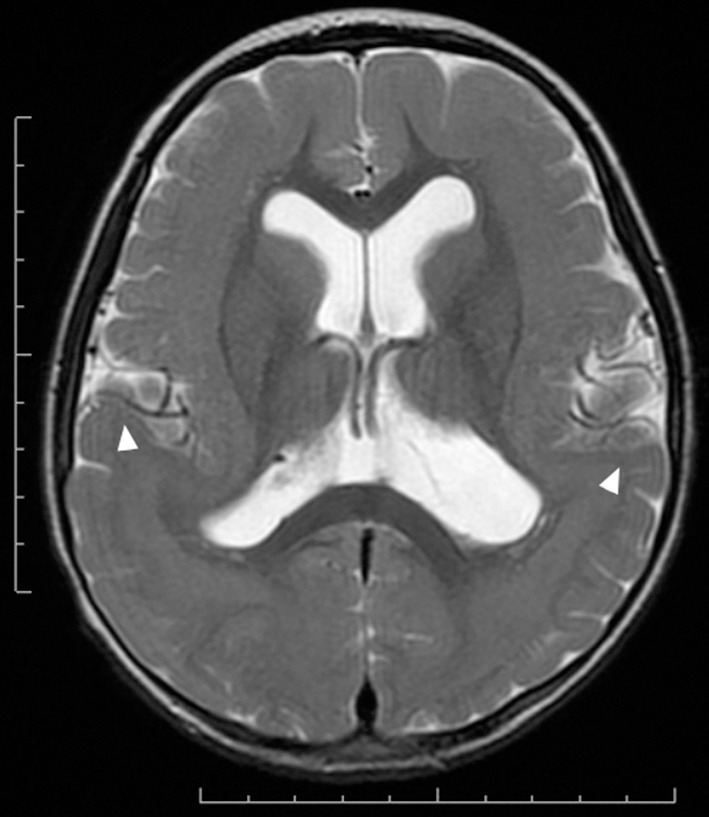
**Subcortical band heterotopia or double cortex syndrome in a** ***DCX*** **mutation patient (Grade 5 on the severity scale)**. T2-weighted axial MRI image. Subcortical heterotopic gray matter in the posterior region fuses into the pachygyric cortex in the anterior region (arrowheads).

**Figure 4:**
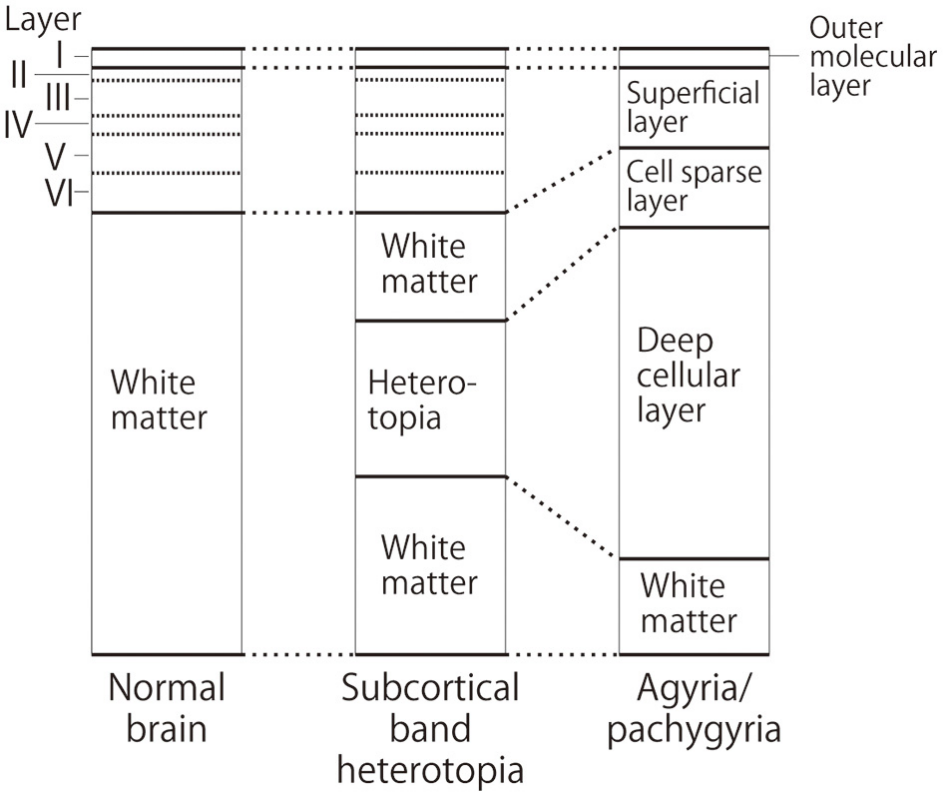
**Schematic diagram of cortical layers in the lissencephaly spectrum compared to the normal brain**. Deep cellular layer of the pachygyric or agyric cortex fuses with laminar or band heterotopia in the subcortical white matter, but not with normal six-layered cortex.

MRI of the brain is useful to discriminate agyria, pachygyria, and subcortical band heterotopia. Agyria is generally characterized by the disappearance of deep sulci in more than one lobe and the thickness of the cortex is 10–20 mm (Figure 1). The gyri in pachygyria are wider than in the normal cortex and the thickness of the cortex is 4–9 mm (Figure 2). Brain MRI of subcortical band heterotopia shows bilateral continuous symmetric bands of gray matter underlying an almost normal cortical mantle with relatively shallow sulci (Figure 3). More than 90% of patients with subcortical band heterotopia are female and the cause is usually heterozygous *DCX* mutation. Subcortical band heterotopia in male patients is caused by somatic mosaic *DCX* mutations or *LIS1* mutations (Gleeson et al., 2000; Kato et al., 2001; D'agostino et al., 2002; Poolos et al., 2002). Coexistence of agyria and pachygyria or pachygyria and subcortical band heterotopia can be seen in the same patient, suggesting common mechanisms for these phenotypes. Microscopically, agyria and pachygyria present a four-layered cortex with an outer molecular layer, superficial layer, cell sparse layer, and deep cellular layer. In the marginal zone between pachygyria and subcortical band heterotopia, the outer molecular layer corresponds to layer I of the normal six-layered cortex, the superficial layer corresponds to layers II–VI, the cell sparse layer corresponds to subcortical white matter, and the deep cellular layer corresponds to band heterotopia with a mass of unlayered ectopic neurons (Figure 4). The primary pathology of lissencephaly due to the *DCX* mutations shows only minor differences compared with that caused by *LIS1* mutations, for example, inferior olivary ectopia is present in *LIS1* mutation brains but is absent in *DCX* mutation brain (Berg et al., 1998); however, Viot et al. report a different cortical architecture for *DCX* lissencephaly (Viot et al., 2004).

The severity of the clinical manifestations of lissencephaly spectrum is correlated with the degree of brain malformation. Patients with agyria show severe muscle hypotonia from infancy (known as floppy infant) and achieve neither head control nor are they able to say meaningful words. A specific form of epileptic seizure, epileptic spasms, occurs in 80% of patients with agyria or pachygyria, although electroencephalography (EEG) may not present with typical hypsarrhythmia, which is characteristically seen in infantile spasms or West syndrome (Guerrini, 2005). However, the main clinical features of subcortical band heterotopia are intellectual disability and epileptic seizures, both of which are milder than those of agyria or pachygyria. Intellectual disability ranges from normal to severe retardation and correlates with the thickness of the band and the degree of pachygyria (Barkovich et al., 1994; Bahi-Buisson et al., 2013). Genetic counseling is particularly important for parents that have a boy with classical lissencephaly or a girl with subcortical band heterotopia because the mother may be a heterozygous carrier of the *DCX* mutation.

Miller-Dieker syndrome is a contiguous gene syndrome caused by a microdeletion in 17p13.3, a region that contains *LIS1* and *YWHAE* (which encodes 14-3-3 protein epsilon). Phenotypes of Miller-Dieker syndrome are more severe than that of classical lissencephaly because of an isolated *LIS1* mutation. They are characterized by complete agyria and facial abnormalities including prominent forehead, bitemporal hollowing, short nose with upturned nares, prominent upper lip with downturned vermilion border and small jaw, and sometimes other congenital defects involving the heart, kidneys, intestine, or fingers (Kato and Dobyns, 2003). Neurological findings of Miller–Dieker syndrome are similar to those of patients with agyria, such as severe developmental delay with weak muscle tone and profound intellectual disability, intractable seizures, dysphagia, and poor prognosis with recurrent infection of the respiratory system.

## Tubulin-related disorders, tubulinopathies

**Figure 5:**
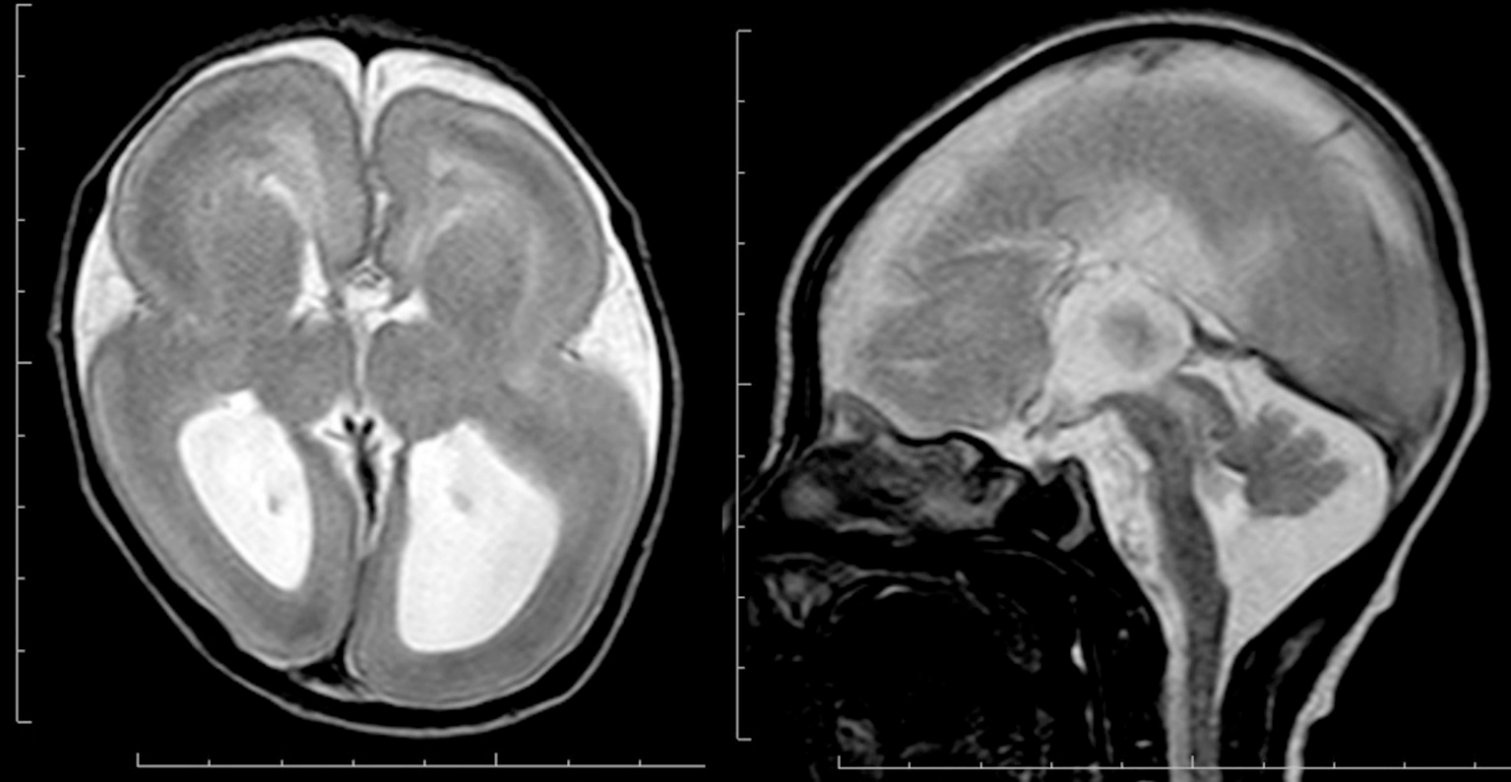
**Complete agyria in a** ***TUBA1A*** **mutation patient (Grade 1 on the severity scale)**. T2-weighted axial MRI image (left) and midsagittal image (right). The boundary of the caudate nucleus and lentiform nucleus is obscure. Complete agenesis of the corpus callosum and pontocerebellar hypoplasia are also seen.

Microtubules provide the main structural framework for the shafts of axons and dendrites, and with actin serve as tracks for intracellular trafficking and to provide the driving force underlying neurite extension and intracellular movement of organelles during mitosis (Flynn et al., 2013). Recently, genes involved in microtubule function have been identified to be causative for various human diseases, such as lissencephaly (Keays et al., 2007; Poirier et al., 2007), polymicrogyria (Abdollahi et al., 2009; Jaglin et al., 2009; Jansen et al., 2011), simplified gyral patter in which the cortical thickness is normal (Cushion et al., 2014), complex brain malformations (Poirier et al., 2010, 2013; Breuss et al., 2012), abnormal eye movement (Tischfield et al., 2010), torsion dystonia (Hersheson et al., 2013), and hypomyelinating leukodystrophy (Simons et al., 2013). All the above are classified as tubulinopathies (Cushion et al., 2013; Bahi-Buisson et al., 2014). Microtubules are assembled from soluble tubulin heterodimers consisting of alpha- and beta-tubulin. Multiple isoforms of both tubulins are encoded by different genes. Mutations of *TUBA1A*, which encodes alpha tubulin, cause lissencephaly spectrum, particularly diffuse agyria or perisylvian pachygyria, with microcephaly, agenesis of the corpus callosum, and cerebellar hypoplasia (Figure 5) (Bahi-Buisson et al., 2008). *TUBA1A* mutations account for only 1% of isolated classical lissencephaly; however, they account for approximately 30% of patients with lissencephaly associated with cerebellar hypoplasia (Kumar et al., 2010). Dysgenesis of the anterior limb of the internal capsule and disorganization of the hippocampus are other neuroimaging features for *TUBA1A* mutation (Poirier et al., 2007). Mutations of *TUBA1A* cause polymicrogyria as well. Interestingly, mutations of *TUBB2B* cause polymicrogyria with or without congenital fibrosis of the external ocular muscles as well as bilateral perisylvian pachygyria(Cederquist et al., 2012; Romaniello et al., 2014). Polymicrogyria is classified as a group of malformations that appear secondary to post-migration development; however, recent findings of the underlying molecular mechanisms reveal overlapping process in neuronal migration and post-migration development stages.

Mutations of *TUBA8* cause polymicrogyria with optic nerve hypoplasia and display autosomal recessive inheritance (Abdollahi et al., 2009). Mutations of *TUBB2A*, which encodes beta-tubulin, cause infantile-onset epilepsy with simplified gyral patterning (Cederquist et al., 2012; Cushion et al., 2014; Romaniello et al., 2014). Mutations of *TUBB3* cause two distinct forms. One is congenital fibrosis of the external ocular muscles or oculomotor nerve hypoplasia and later-onset peripheral axon degeneration with dysgenesis of the corpus callosum, anterior commissure, and internal capsule, but with no cortical dysplasia suggesting migrational defects (Tischfield et al., 2010). Another is cortical dysgenesis including polymicrogyria, pontocerebellar hypoplasia, and abnormal basal ganglia, but with no ocular motility defects (Poirier et al., 2010). The main mechanisms underlying the phenotypes caused by*TUBB3* mutations are impaired axon guidance owing to disrupted microtubule dynamics and kinesin interaction (Tischfield et al., 2010). Tubulinopathies caused by the mutations of the genes encoding alpha- or beta-tubulin demonstrate more extensive phenotypes compared to other gene mutations, such as *LIS1, DCX*, or *RELN*. Mutations of *TUBA1A*, which encodes alpha-tubulin 1A, is the most frequently found in patients with brain malformations, while more genes encoding beta-tubulin, such as *TUBB2A, TUBB2B, TUBB3, TUBB4A*, and *TUBB*, are identified in a wide spectrum of disorders besides brain malformations. Pathological mechanisms and discrepancy between alpha- and beta-tubulinopathies should be elucidated.

## ARX-related disorders, interneuronopathies

The embryonic cerebral cortex at the stage of neuronal migration contains neuronal cells with two modes of migration; radial migration from the ventricular zone toward the pia and tangential migration from ganglionic eminence along a tangential trajectory into the developing cortex. Radially migrating neurons in the cortex are mainly excitatory projection neurons expressing glutamate as a neurotransmitter. Tangentially migrating neurons are inhibitory interneurons expressing the neurotransmitter GABA. XLAG is caused by mutation of *ARX*, which is expressed in the embryonic ganglionic eminence, neocortex, and hippocampus and plays important roles in neuronal proliferation, interneuronal migration, and differentiation in the embryonic forebrain, as well as a secondary role in differentiation of the testes (Kitamura et al., 2002). Patients with XLAG present occipital-predominant classical lissencephaly, particularly anterior pachygyria and posterior agyria, or a simplified gyral pattern, agenesis of the corpus callosum, and abnormal basal ganglia (Kato et al., 2004). In the most severe form of XLAG, patients show hydranencephaly with a large occipital cavity. Female carriers of *ARX* mutations causing XLAG have a risk of agenesis of the corpus callosum with no cortical defects. Abnormalities of external genitalia range from hypoplastic penis or undescended testes to complete female appearance, while the karyotype is 46,XY. Neuropathological studies show a complete loss or a decreased number of cortical interneurons in human XLAG and in *Arx*-null mice (Bonneau et al., 2002; Kitamura et al., 2002) and a three-layered cortex in human XLAG (Forman et al., 2005). Patients with XLAG show intractable seizures soon after birth, suggesting a great disparity between excitatory projection neurons and inhibitory interneurons. *ARX* mutations in patients with XLAG are null mutations or non-conservative missense mutations at critical amino acids in the homeodomain, while other missense mutations or expansion mutations in the polyalanine tract result in X-linked intellectual disability with or without dystonia, West syndrome, Ohtahara syndrome, or early infantile epileptic encephalopathy with suppression burst on EEG but with no brain malformation (Bienvenu et al., 2002; Stromme et al., 2002; Guerrini et al., 2007; Kato et al., 2007, 2010). Interestingly, longer polyalanine expansion is correlated with more severe and earlier onset phenotypes. A wide spectrum of ARX-related disorders forms a group of interneuronopathies based on the role of ARX during neurogenesis, as seen in patients and in the *Arx*-null mouse model (Kato and Dobyns, 2005; Marsh et al., 2009).

## Classical lissencephalies associated with other forms of brain malformation

Classical lissencephaly caused by *LIS1* or *DCX* mutations usually exist in isolated forms and only show cortical dysplasia on brain MRI. Rare variant forms of lissencephaly are associated with congenital microcephaly, cerebellar hypoplasia, or agenesis of the corpus callosum. Each form demonstrates characteristic radiological findings and some of the causative genes have been identified.

A lissencephaly group with cerebellar hypoplasia can be classified into several types according to brain imaging, additional clinical features, and causative genes (Ross et al., 2001). Among them, frontal predominant mild lissencephaly (diffuse pachygyria) with severe hippocampal and cerebellar hypoplasia or Reelin-type lissencephaly is caused by mutation of either *RELN* or *VLDLR* and shows autosomal recessive inheritance (Hong et al., 2000; Boycott et al., 2005). Dysequilibrium syndrome is an allelic disorder of the *VLDLR* locus (Moheb et al., 2008). Reelin-type lissencephaly has an inverted or no clear pattern of cortical lamination attributable to abnormal migration of the neurons in an outside-in birth order (Cooper, 2008; Dekimoto et al., 2010).

Lissencephaly can be associated with congenital microcephaly, though the head circumference of lissencephaly caused by the *LIS1* or *DCX* mutations is usually within the normal range. Lissencephaly with a head circumference of less than −3 SD at birth is classified as microlissencephaly (Barkovich et al., 2005) or microcephaly with lissencephaly (Barkovich et al., 2012). Although many genes identified to be responsible for primary microcephaly, such as *MCPH1, ASPM, CENPJ, CDK5RAP2*, and *PNKP*, are involved with the cell-cycle phase of mitosis affecting neurogenesis (Barbelanne and Tsang, 2014), the causative genes for microlissencephaly remain unknown in many cases. Mutations of *WDR62*, which encodes a protein localized to centrosomes throughout mitosis and nucleoli during interphase, cause microcephaly with pachygyria or polymicrogyria (Bilguvar et al., 2010). Mutations of *NDE1*, which encodes a protein that binds dynein and functions in centrosome duplication, as well as the *TUBA1A* mutations mentioned above, cause microcephaly with a simplified gyral pattern, agenesis of the corpus callosum, and cerebellar hypoplasia (Alkuraya et al., 2011; Bakircioglu et al., 2011).

## Conclusion

Neuronal migration disorders are classified based on causative genes as well as on brain MRI and neuropathological findings. There are strong relationships between clinical manifestations and mutation of a particular gene, in accordance with the expression and functions of that gene. Recent advances in gene and genome analysis technology will enable the genetic basis of neuronal migration disorders to be readily determined, facilitating the elucidation of genotype-phenotype correlations.

### Conflict of interest statement

The author declares that the research was conducted in the absence of any commercial or financial relationships that could be construed as a potential conflict of interest.
